# Reproductive justice in the time of COVID-19: a systematic review of the indirect impacts of COVID-19 on sexual and reproductive health

**DOI:** 10.1186/s12978-021-01286-6

**Published:** 2021-12-20

**Authors:** Trena I. Mukherjee, Angubeen G. Khan, Anindita Dasgupta, Goleen Samari

**Affiliations:** 1 Department of Epidemiology, Mailman School of Public Health, Columbia University, New York, NY USA; 2 Department of Community Health Sciences, Fielding School of Public Health, University of California Los Angeles, Los Angeles, CA USA; 3 Social Intervention Group, School of Social Work, Columbia University, New York, NY USA; 4 Heilbrunn Department of Population and Family Health, Mailman School of Public Health, Columbia University, New York, NY USA

**Keywords:** COVID-19, Reproductive health, Sexual health, Gender, Health inequity, Abortion, Contraceptives

## Abstract

**Objective:**

Despite gendered dimensions of COVID-19 becoming increasingly apparent, the impact of COVID-19 and other respiratory epidemics on women and girls’ sexual and reproductive health (SRH) have yet to be synthesized. This review uses a reproductive justice framework to systematically review empirical evidence of the indirect impacts of respiratory epidemics on SRH.

**Methods:**

We searched MEDLINE and CINAHL for original, peer-reviewed articles related to respiratory epidemics and women and girls’ SRH through May 31, 2021. Studies focusing on various SRH outcomes were included, however those exclusively examining pregnancy, perinatal-related outcomes, and gender-based violence were excluded due to previously published systematic reviews on these topics. The review consisted of title and abstract screening, full-text screening, and data abstraction.

**Results:**

Twenty-four studies met all eligibility criteria. These studies emphasized that COVID-19 resulted in service disruptions that effected access to abortion, contraceptives, HIV/STI testing, and changes in sexual behaviors, menstruation, and pregnancy intentions.

**Conclusions:**

These findings highlight the need to enact policies that ensure equitable, timely access to quality SRH services for women and girls, despite quarantine and distancing policies. Research gaps include understanding how COVID-19 disruptions in SRH service provision, access and/or utilization have impacted underserved populations and those with intersectional identities, who faced SRH inequities notwithstanding an epidemic. More robust research is also needed to understand the indirect impact of COVID-19 and epidemic control measures on a wider range of SRH outcomes (e.g., menstrual disorders, fertility services, gynecologic oncology) in the long-term.

## Plain English summary

The impact of respiratory epidemics, like COVID-19 on women and girls’ sexual and reproductive health (SRH) is not yet known. This review applies a reproductive justice framework, to systematically review the impact of respiratory epidemics on SRH, in order to examine the impact of COVID-19 on equitable, sustained access to quality SRH services for all populations. This framework highlights the right to reproductive autonomy, including the right to have an abortion, conceive, bear and raise children; and is inclusive of the intersectionality of race, class and gender. This review includes original, peer-reviewed research related to COVID-19 and women and girls’ SRH through May 31, 2021, and consisted of title and abstract screening, full-text screening, and data abstraction. Overall, twenty-four studies met eligibility criteria. Results emphasize that the COVID-19 pandemic resulted in service disruptions that effected access to abortion, contraceptives, HIV/STI testing, and changes in changes in sexual behaviors, menstruation, and pregnancy intentions. These findings highlight the urgent need to enact policies that ensure equitable, timely access to quality SRH services for women and girls, despite pandemic response policies. This review also highlights opportunities to better understand how COVID-19 related disruptions in SRH service provision, access and/or utilization have impacted underserved populations and those with intersectional identities, who faced SRH inequities prior to the COVID-19 pandemic. More research is needed to understand the indirect impact of COVID-19 and epidemic control measures on a wider range of SRH outcomes (e.g., menstrual disorders, fertility services, gynecologic oncology) in the long-term.

## Background

As the COVID-19 pandemic continues to take lives worldwide, an understanding of the short- and long-term consequences of the pandemic on women’s and girls’ sexual and reproductive health (SRH) is critical [1, 2]. Global responses, lockdowns, and travel restrictions converge with pervasive, existing health inequities and injustices to disproportionately impact the health, wellbeing, and economic stability of women and girls [3]. The indirect consequences of COVID-19 control may be overlooked in the immediate need to mitigate transmission, and SRH-related morbidity and mortality will not become apparent for years to come. Several commentaries have discussed the disruptions to SRH care provision that providers and family planning clinics experienced [1, 2, 4, 5]; including interruptions to the supply and provision of contraception, abortion and post-abortion care, a decline in the number of patients served due to inaccessibility, and reduced client engagement as lockdowns and travel restrictions went into effect [6].

In light of the efforts to exclude SRH from essential health services during COVID-19 [1–3, 7], an understanding of the impact of the COVID-19 pandemic on SRH is critical for informing future actions and policies that prevent adverse SRH outcomes and comorbidities. Evidence from the SARS, MERS and Ebola pandemics envisage that the populations for whom human rights are least protected and most violated (e.g. women/girls, youth, poor people, immigrants, racial/ethnic minorities) will experience severe, unique difficulties and differentially die from COVID-19 [8, 9]. Even prior to the COVID-19 pandemic, African American/Black, Latinx, immigrant, and women and girls with lower socioeconomic status experienced greater SRH disparities [10–13]. The restrictions on movement disproportionately affect marginalized populations, and simulation studies estimate that COVID-19 related disruptions in essential SRH care will result in declines in short- and long-acting reversible contraceptive use, and increases in unintended pregnancies and unsafe abortions [14]. Conservative estimates of the impact of service disruptions at Marie Stopes International-affiliated health facilities across 37 countries suggest that the COVID-19 pandemic could result in 1.3 million unintended pregnancies, 1.2 million unsafe abortions, and 5000 pregnancy-related deaths [6]. Therefore, it is crucial to apply a reproductive justice framework to ensure equitable, sustained access to quality SRH services for all populations throughout the duration of the COVID-19 pandemic. This framework highlights the right to reproductive autonomy, including the right to have an abortion, and to conceive, bear and raise children; and is inclusive of the intersectionality of race, class and gender [15, 16].

Despite hypothesized impacts, empirical evidence of the indirect impacts of the COVID-19 pandemic on women and girls SRH have yet to be synthesized. We apply a reproductive justice framework to systematically review empirical evidence on the indirect impacts of the COVID-19 pandemic on women and girls’ SRH, in order to identify the observed effects of COVID-19 and the pandemic response on SRH; and to highlight SRH disparities for marginalized women and girls who are all too often overlooked and underserved.

## Main text

### Methods

A protocol with search terms was developed in consultation with and approved by a trained systematic review specialist at Columbia University. Respiratory illness related search terms included “pandemic, epidemic, outbreak, influenza, COVID-19, coronavirus, 1918 Flu, Middle Eastern Respiratory Syndrome, MERS, Severe Acute Respiratory Syndrome, SARS, Swine Flu, and H1N1.” Outlined by the reproductive justice framework [15, 16], with a focus on reproductive autonomy, including the right to have an abortion, and to conceive, bear and raise children, SRH search terms included “preventative and curative care related to pregnancy, fertility, contraception, sexually transmitted infection (STI), reproductive cancers and other reproductive morbidities, gender-based, gender inequities, women’s health, sexual health, reproductive health, obstetric, gynecol*, pregnancy, fertility, contracepti*, abortion, family planning, STI/STD, sexual violence, maternal health, reproductive coercion, maternal mortality, reproductive justice, menstrual hygiene, and reproductive tract infection.”

Peer-reviewed studies published until May 31, 2021 were included from journals across MEDLINE via PubMed and CINAHL (PsychINFO, Gender Studies Database, Violence & Abuse, Women’s Studies International). Inclusion criteria included respiratory illness epidemic and an outcome explicit to women and girls’ SRH. Populations could have been diagnosed with, exposed to, or impacted by public health responses (i.e., service disruptions, lockdowns, etc.) to respiratory epidemics or pandemics. Studies also had to have abstracts, full-texts and be published in a peer-reviewed journal. Articles without English translation, opinion pieces, commentaries, guidelines and simulation/modelling studies were excluded. Those addressing non-respiratory epidemics (i.e., obesity, opioid, HIV, etc.) and those that failed to examine SRH outcomes beyond vaccine interest and/or the psychological and emotional impact of the pandemic among pregnant women were excluded. Although pregnancy and birth-related outcomes, gender-based violence, and maternal and child health fall within the realm of reproductive justice, studies exclusively examining these outcomes were excluded, as systematic reviews including these topics have been recently published elsewhere [17–28]. Studies could have been published in any country including low, middle, and high-income settings, and there was no restriction on study publication dates.

The review consisted of screening: (1) titles, (2) abstracts, (3) full-texts, (4) data abstraction, and (5) critical appraisal of study bias. Each phase was completed independently by study authors. Title, abstract, and full text screening of eligible articles were completed by TM, AK, AD, and GS. Study data (author, study type, epidemic, SRH outcome and major findings) were abstracted by AK and TM. Data-screening procedures were applied according to the eligibility criteria. At the data abstraction stage, reviewers used data collection forms to capture the primary epidemic and primary outcome measure(s), in addition to supplementary information on study design, sampling/data sources, analytical methods, and effect estimates. Studies that met all eligibility criteria were assessed for methodological quality and risk of bias using the Quality Assessment Tool for Studies with Diverse Designs (QATSDD) as it enables review of studies with similar research questions, but different study designs. The QATSSD has shown good reliability and validity for quantitative and qualitative study designs [29, 30]. The QATSDD consists of 16-items (4-items are for quantitative or qualitative studies only) that are rated on a 4-point Likert Scale. Total scores range from 0 to 42, with higher scores indicating higher quality research. Scores were converted into a percentage, and those scoring > 60% were rated as high-quality studies, whereas those scoring ≤ 60% were rated as lower quality studies [31]. TM and AK independently reviewed and rated each study for risk of bias. Any disagreements were discussed until an agreement was reached.

### Results

**Fig. 1:**
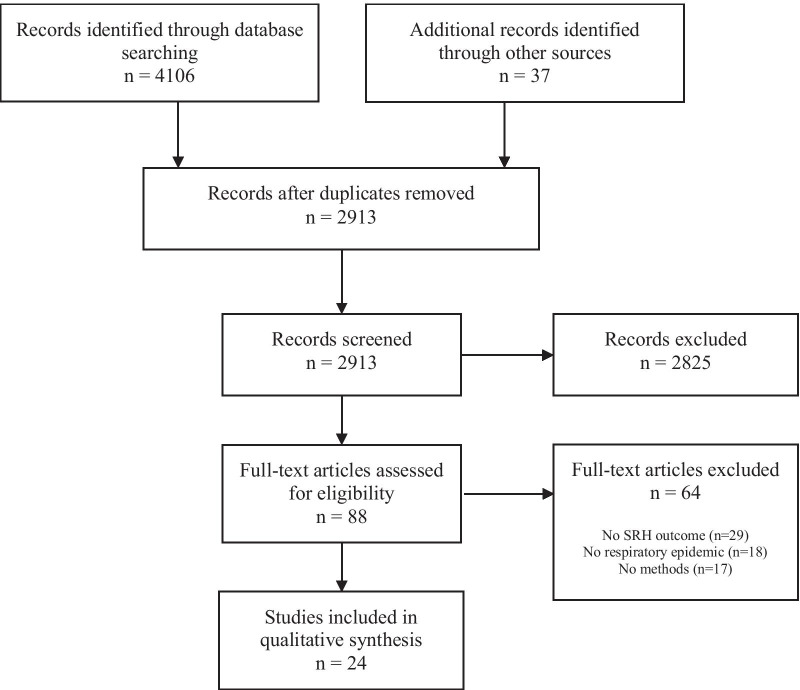
Study selection process

The search returned 2913 unique articles for title and abstract review, of which 88 met eligibility criteria and were included for full text review (Fig. 1). Twenty-four articles met all eligibility criteria after full review (Tables 1, 2). Most (n = 22, 92%) were quantitative, with over half using cross-sectional (n = 13, 55%) study designs. The majority of studies were published in the global North (n = 16, 67%), and all examined the impact of COVID-19. No studies examined the impact of the 1918 Flu, H1N1 and SARS or Middle Eastern Respiratory Syndrome (MERS) on non-pregnancy related SRH outcomes. One-third of studies examined SRH outcomes related to abortion (n = 8, 33%), one-quarter examined changes in service provision (n = 6, 25%), while others examined contraceptive access or utilization (n = 5, 21%), sexual behavior (n = 4, 17%), pregnancy intentions (n = 3, 13%), and menstrual cycle changes (n = 2, 8%). The majority (n = 14, 58%) of studies were rated as having low methodological quality based on the total QATSDD score, with total scores ranging from 31 to 88%.

**Table 1:** Summary of studies included (N = 24)

Characteristic	n (%)
Study design	
Quantitative	22 (91.7)
Cross-sectional	13 (54.2)
Longitudinal	3 (12.5)
Quasi-experimental	2 (8.3)
Retrospective	4 (16.7)
Mixed methods	2 (8.3)
Sample size	
None given	1 (4.2)
1–100	7 (29.2)
101–1000	10 (41.6)
> 1000	6 (25.0)
Region (as defined by WHO)	
Africa	1 (4.2)
Americas	11 (45.8)
South-East Asia	1 (4.2)
Europe	5 (20.8)
Eastern Mediterranean	0 (0.0)
Western Pacific	2 (8.3)
Global	2 (8.3)
Respiratory epidemic	
COVID-19	24 (100)
Primary SRH outcome^a^	
Abortion	8 (33.3)
Contraceptive access/utilization	5 (20.8)
Menstruation	2 (8.3)
Service provision	6 (25.0)
Sexual behavior	4 (16.7)
Pregnancy intentions	3 (12.5)
QATSDD	
High	10 (41.7)
Low	14 (58.3)

*SRH* sexual and reproductive health; *COVID-19* coronavirus disease 2019, *WHO* World Health Organization; *QATSDD* Quality Assessment Tool for Studies with Diverse Designs

^a^May not equate to 100% due to multiple outcomes

**Table 2:** Study characteristics

Author(s)	Methods	Sample	Epidemic	SRH outcome	Major findings
Aiken et al. [32]	Quantitative; Quasi-experimental	Individuals seeking online abortion telemedicine services in the US between Jan 1, 2019 and April 11, 2020 (n = 49,935)	COVID-19	Abortion	• From March 20–April 11, 2020 ('after' COVID-19), there was a 27% increase in requests for self-managed medication abortion across the US • States with significant increases in requests had higher rates of stay-at-home-behaviors, especially high rates of COVID-19, and/or more severe COVID-19 related restriction on in-clinic abortion access
Aryal et al. [39]	Quantitative; Cross-sectional	Women provided with safe abortion services between April—June 2020 (lockdown period) and July–September 2020 (lockdown eased) in western Nepal (n = 52)	COVID-19	Abortion	• COVID-19 pandemic related lockdowns reduced the number of women coming in for abortions by 25.7%. Additionally, 47.1% more women came in for an abortion later in the pandemic, when pandemic related restrictions eased, compared to earlier in the pandemic, with strict lockdowns • Women who came in for an abortion earlier in the pandemic had a later period of gestation compared to women who came in when restrictions eased (9.5 weeks vs 7.5 weeks; p = 0.049). Distance from health facility was also significantly associated with accessibility to the health facility, with women who lived more than 5 h from the facility not seeking an abortion early in the pandemic (p = 0.021) • Finally, 48% of all women enrolled in the study reported an increased need for contraception, with 23% of women not using contraceptives due to inaccessibility due to lockdowns
Caruso et al. [43]	Quantitative; Cross-sectional	Women who use hormonal contraceptives and were registered at a family planning clinic in Italy (n = 317)	COVID-19	Contraceptive utilization	• Two-thirds of participants used short-acting reversible contraceptives (SARC; oral contraceptive pill = 53.3%; vaginal ring = 14.2%) and one-third of participants used long-acting reversible contraceptives (LARC; subdermal implant = 19.2%); IUD = 13.2%) • 70% of women used contraceptives to avoid unplanned pregnancies; 30% used contraceptives for additional non-contraceptive benefits • Women who married or co-habiting continued to use contraceptives and had no unplanned pregnancies • Half of all women (n = 51) who were not co-habiting discontinued SARC; half continued to engage in sexual activity (n = 47) and 15% (n = 15) had an unplanned pregnancy, for which they sought abortion
Coombe et al. [46]	Mixed methods; Cross-sectional	Australian women of reproductive age (18–49 years) (n = 518)	COVID-19	Pregnancy intentions & access to contraceptives	• Most participants (76%) indicated that they were trying to avoid pregnancy • Nearly 20% of women were not using any contraception. Of those that were, the oral contraceptive pill was the most common (21%) method • When asked about SRH access during lockdown, only 9% reporting difficulties accessing contraception. For some this was due to shortages in their preferred method, difficulty obtaining a doctor’s appointment, fear of leaving the house due to COVID-19, or privacy concerns. Women that were unemployed reported greater difficulty accessing contraceptives (OR: 2.5 (1.1- 5.0)). Nearly a quarter (22%) of women reported needing to access SRH-related healthcare, with little difference by socio-demographics • Most participants indicated that the COVID-19 pandemic had not changed their pregnancy intentions, however, some women indicated that they had actively stopped trying during the pandemic, or that they were unable to continue trying to conceive due to cancellations of IVF or other reproductive services
Dell'Utri et al. [50]	Quantitative; Retrospective cohort	Medical records data of women admitted for obstetric and gynecological emergency services at the largest maternity clinic in Milan, Italy between Feb 23-June 24 in 2019 and 2020 (N = 9291)	COVID-19	Obstetric and gynecological service provision	• Compared to the reference year (2019), registered admissions during stay-in-place measures in 2020 decreased by 35.4% (34.1 to 36.6), with the highest reduction corresponding to the maximum increase of newly infected cases • The decrease was nearly double among Italians, compared to foreign women, with no decrease observed among African women • There was a 63.5% (60.5 to 66.5) reduction in gynecological complaints, particularly for admissions for vulvovaginal infections, urogynecological conditions and/or cystitis (− 75.7% (71.4 to 80.1); menorrhagia/atypical blood loss (− 41.4% (− 31.7 to 51.1); and pelvic inflammatory disease (− 61.5% (− 35.1 to 88.0)
Endler et al. [38]	Quantitative; Cross-sectional	SRH-related clinicians, researchers and practitioners from 29 countries (n = 51)	COVID-19	Contraceptive access & abortion	• Nearly all (86%) reported that access to contraceptives decreased during the COVID-19 pandemic, whereas 62% and 46% of respondents reported decreases in surgical and medical abortion, respectively • The highest perceived barriers to abortion access were fear of COVID-19 infection, lack of transportation, and closed pharmacies. Most respondents indicated that SRHR services decreased due to the prioritization of the COVID-19 pandemic response, or that the pandemic was an excuse to pause, ignore or dismantle any progress made toward advancing SRHR. A few exceptions were made in high-income countries, where the pandemic provided an opportunity to advance access to SRHR • Compared to countries with mildly restrictive abortion policies, countries with severely restrictive abortion policies reported less women accessing SRH-related services (69% vs 23%; p = 0.026); no abortion policy changes (69% vs 0%); p < 0.001); and decreased contraceptive policy changes (88% vs 46%; p = 0.023)
Fuchs et al. [53]	Quantitative; Longitudinal	Sexually active women between the ages of 18–40 years (n = 764) in Poland	COVID-19	Sexual behavior	• Total sexual function as measured by the Female Sexual Function Index (FSFI) significantly decreased (30.1 (4.4) vs 25.8 (9.7)) during the COVID-19 pandemic. Decreases were seen across every FSFI domain (desire, arousal, lubrication, orgasm, satisfaction, and pain) • Sexual dysfunction (FSFI score < 26) increased during COVID-19 control measures (15.3% vs 34.3%) • Frequency of sexual intercourse declined due to isolation, conflict with partner and mental health (stress, anxiety, depression) • Less educated women, those with worse living conditions, women who did not work, women living with their parents or those in informal relationships experienced lowest sexual functioning
Kerestes et al. [34]	Quantitative; Retrospective cohort	Patients who had medication abortion up to 77 days gestation between April and November 2020 in Hawaii (n = 334)	COVID-19	Abortion	• A total of 334 patients received medication abortion, of which 149 (45%) received telemedicine with in-person pickup of medications, 75 (23%) received telemedicine with medications mailed, and 110 (33%) received traditional in person visits • The rate of complete medication abortion without surgical intervention was 96%, with success rates of 97%, 97%, and 94% for the clinic pickup, mail, and clinic visit groups, respectively • Success rate for those with and without an ultrasound prior were similar (96% vs 97%), and 88% of patients returned for follow up care. Very few women (17; 5%) experienced any complications
Leight et al. [42]	Quantitative; Quasi-experimental	n = 109,129 women served by n = 132 unique promoters (community healthcare workers) and 192 unique public health facilities in Nampula and Sofala, Mozambique between January 21—May 20, 2020	COVID-19	Access to contraceptives	• COVID-19 related lockdowns and disruptions were associated with a decrease in contraceptive receipt (OR 0.798, 95% CI [0.701–0.908], p = 0.001) • Easing lockdown restrictions was associated with an increase in contraceptive referrals (OR 1.187, 95% CI [1.034, 1.354], p = 0.015), especially amongst women who were not currently using contraceptives (OR 1.490, 95% CI [1.203, 1.841], p < 0.001); and in contraceptive receipt (OR 0.777, 95% CI [0.660, 0.913], p = 0.002), especially among women with phone access (OR 1.800, 95% CI [1.469, 2.205], p < 0.001)
Li et al. [45]	Quantitative; Cross-sectional	Young citizens between the ages of 18–35 years who reported having sexual intercourse in the 6 months prior (n = 967) in China	COVID-19	Sexual behavior & service provision	• COVID-19 control measures resulted decrease in sexual desire (20%), frequency of sex (41%), alcohol consumption before or during sexual activities (20%), and risky sexual behavior (10%), partner deterioration (31%) • Partner relationships were influenced by housing (aOR: 0.59; 95% CI 0.30–0.86), exclusivity (aOR 0.44; 95% CI 0.27–0.73); sexual desire (aOR 2.01; 95% CI 1.38–2.97); and sexual satisfaction (aOR 1.92; 95% CI 1.54–2.50) • Although the numbers were small, participants reported difficulties in accessing reproductive health services: Women with recent abortions described difficulties making appointments Participants with STIs described difficulties obtaining a doctor’s appointment and in accessing medications 8.9% reported experiencing a shortage of contraceptives
Lin et al. [47]	Quantitative; Cross-sectional	Women between the ages of 18–49 year who reside in the US between May 16 and June 16, 2020 (n = 554)	COVID-19	Pregnancy intentions & access to contraceptives	• Compared to White respondents, Latinx (OR 4.01 (2.25–7.15)), Black/African Americans (OR 3.92 (1.81–8.50)) and Multiracial (OR 2.12 (1.10–4.07)) respondents reported higher odds of inability to afford food, transportation, and/or housing during the pandemic; and Hispanics/Latinx [1.95 (1.12–3.40)] reported higher odds of food insecurity • Inability to afford food, transportation, and/or housing was associated with a decreased desire for pregnancy [OR 2.13 (1.32–3.43)], and greater difficulty accessing contraceptives (OR 1.86 (1.06–3.24)
Luetke et al. [54]	Quantitative; Cross-sectional	Nationally representative weighted sample of partnered men and women (n = 742) in the US	COVID-19	Sexual behaviors	• One-third of participants (34%) reported some degree of COVID-related conflict with their romantic partners • Compared to those experiencing no conflict, those with any conflict reported decreased odds of intimate behavior: hugging, kissing, holding hands, cuddling (aOR: 2.35, 95% CI 1.58–3.50); giving/receiving oral sex (aOR: 2.34, 95% CI 1.36–4.02); intercourse (aOR: 2.28, 95% CI 1.40, 3.73) • There was a dose–response curve between conflict and intimate/sexual behaviors; and those that reported more conflict exhibited less sexual behaviors
Mello et al. [37]	Mixed methods; Policy analysis & Cross-sectional quantitative survey	Abortion health facilities in Ohio, Kentucky & West Virginia (n = 14)	COVID-19	Abortion	• Despite federal regulations encouraging the utilization of medication abortions in the US, state laws governing medication and telemedicine abortion in Ohio, Kentucky and West Virginia remained in effect throughout 2020 and barred patients from receiving medication abortions by mail • Surveys with abortion facilities indicate that an average of 2107 abortions were performed monthly between December 2019 and December 2020, 42% (n = 893) of which were medication abortions • Coinciding COVID-19 pandemic related disruptions and executive orders, the number of total (n = 2306) and medication abortions (n = 1613; 70%) peaked in April 2020 and returned to pre-pandemic levels by June 2020. The peak is most stark for Ohio (72%, vs 40% average) and West Virginia (87%, vs 49% average); whereas Kentucky sees only a slight increase (55% vs 50% average)
Micelli et al. [55]	Quantitative; Cross-sectional	Italian men and women in long-term relationships between the ages of 18–46 years (n = 1482; n_women_ = 944, n_men_ = 538)	COVID-19	Pregnancy intentions	• Of the 18% participants who were planning to have a child before the pandemic, 37% abandoned the intention because of worries about future economic difficulties (58%) and consequences on pregnancy (58%) • Of 82% who did not intend to conceive, 12% revealed a desire for parenthood during quarantine than before (p < 0.01), related to will for change (50%) and need for positivity (40%). 4.3% of these tried to get pregnant • Stratifying by age, a trend toward older ages was found in the desire for parenthood before and during the COVID-19 pandemic (p < 0.05)
Nagendra et al. [49]	Quantitative; Cross-sectional	Convenience sample of individuals on the NYC STD PTC educational mailing list and key partners from state and local health department (N = 73, n_New York_ = 61, n = 12 from Indiana, Ohio, Michigan, New Jersey, Puerto Rico, or United States Virgin Islands)	COVID-19	Service provision	• Majority of clinics providing sexual health services indicated a significant decrease in the regular services they were able to provide, except for expansion in telehealth services • Only 25% of the clinics that offered pregnancy termination and 18% of clinics (n = 11) that offered STI testing services before March 1, 2020, could do so as of April 1, 2020 • 80% of clinics have resorted to treating STIs presumptively based on symptomology, before testing, due to the COVID-19 outbreak in the US • As of April 1, 2020, only 25% of respondents located in NYS and 26% outside of NYS are able to offer HIV testing
Phelan et al. [48]	Quantitative; Cross-sectional	Women of reproductive age, globally (n = 1031)	COVID-19	Menstruation	• Nearly a quarter (23%) of respondents were using hormonal contraception • Almost half (46%) reported a change in menstrual cycle since the beginning of the pandemic, with 53% reported worsening premenstrual symptoms, 18% reporting new menorrhagia and 30% new dysmenorrhea compared to before the pandemic (p < 0.05) • A small number of respondents (9%) reported missed periods whereas they previously did not (p = 0.003), with a median number of 2 (1–3) missed periods. 21% of those who “occasionally” missed periods pre-pandemic missed periods “often” during pandemic • Nearly half of all women (45%) reported a reduced libido • There was no change in the median cycle length (28 days) or days of bleeding (5) but there was a wider variability of cycle length (p = 0.01) and a 1-day median decrease in the minimum and maximum cycle length (p < 0.05)
Rimmer et al. [51]	Quantitative; Cross-sectional	Junior obstetrics and gynecology doctors across training units in the UK National Health Service UK (n = 148)	COVID-19	Obstetrics and gynecology service provision	• Majority of units (60%) completed training drills for managing obstetrics and gynecology emergencies during COVID-19, nearly all (88%) implemented COVID-19 specific protocols, had adequate PPE (91%), operated dedicated COVID-19 obstetric emergency theatres (71%) • Most had to reduce in-person antenatal clinics (79%), but offered telehealth services (71%) and dedicated clinic areas for pregnant women with confirmed or suspected COVID-19 (78%) • Elective gynecological services (fertility and urogynaecology) were mostly suspended (89%); 40% implemented protocols to reduce inpatient stays, including medical management as the first line of treatment for miscarriage (59%) or ectopic pregnancies (28%) in order to reduce inpatient stays • Oncological referral pathways were unaffected in half (51%) of all units, with planned reductions in oncology surgery in half (55%) of all units • Rapidly changing protocols and lack of clarity led to confusion among doctors
Roberts et al. [35]	Quantitative; Cross-sectional	Providers at independent abortion clinics across the US (n_clinics_ = 103)	COVID-19	Abortion	• Clinics in all regions of the US were represented: Northeast (21%), Midwest (25%), South (31%), and West (22%) • Over half of all clinics (51%) had to clinicians/staff who were unable to work because of the pandemic. Clinicians were unable to provide care because they were quarantined (23%), part of a high-risk group (21%), sick with possible COVID-19 (20%), subject to COVID-19 related travel restrictions (15%, re-assigned to other COVID-19 related responsibilities (13%), or had childcare (12%) or other caregiving (5%) responsibilities • Non-clinical staff reported being unable to work because of childcare responsibilities (50%), being sick with possible COVID-19 (45%), quarantine (44%), belonging to a high-risk group (33%) and having caregiving responsibilities (18%). 40% of respondents reported that they had to cancel or postpone appointments because patients had COVID-19 symptoms or had been exposed, and 13% had patients who were subject to COVID-related restrictions on travel • Most clinics had had to cancel or postpone some clinical services, including gynecologic services (59%), contraceptive visits (55%) and STI tests (45%) • More than 60% of respondents in the Northeast, Midwest and West reported that their state had declared abortion essential, compared to just 14% in the South. 38% of clinics had canceled or postponed first-trimester aspiration abortions, 27% second-trimester or later abortions and 25% medication abortions • The proportion of clinics that had canceled or postponed first-trimester aspiration abortions was highest in the South (66%) and Midwest (38%), compared to 9% Northeast and 26% in the West • 19% reported having to close their clinic temporarily, especially in the Midwest (21%) and South (35%)
Roland et al. [41]	Quantitative; Longitudinal	National Health Data System insurance claims of all pharmacy dispensations of residents of France between January 1, 2018–June 7, 2018, 2019 and 2020	COVID-19	Contraceptive utilization	• Oral contraceptive dispensation increased during the first two weeks of lockdown by 47% and 16%, but thereafter decreased • Overall, the number of prescriptions of oral contraceptives, emergency contraception, intrauterine devices (IUDs), and ovulation indicators decreased over the course of the 8-week lockdown by 46,603; 38, 429; 21,250; and 44,510 respectively • In the 4-weeks post-lockdown, prescriptions continued to decrease, and the number of prescriptions of oral contraceptives, emergency contraception, intrauterine devices (IUDs), and ovulation indicators decreased by 70,021; 11,226; 1,807; and 17,431, respectively
Stifani et al. [40]	Quantitative; Cross-sectional	Family planning providers in the US (n = 172)	COVID-19	Access to contraceptives	• 91% of surveyed providers provided telemedicine services during the COVID-19 pandemic. About half of providers (53%) referred less than a quarter of telemedicine patients to in-person visits, with the most common reason being LARC insertion (53%) • Almost all providers reported that the following services were available to their patients even at the height of the COVID-19 pandemic: LARC insertions (88%); LARC removals (90%); depot-medroxyprogesterone acetate injections (88%), and visits for other contraception-related issues (85%) • Most providers (80%) agreed that telemedicine is an effective way to conduct contraceptive counseling, and that the role of telemedicine for contraceptive counseling should be expanded after the pandemic (84%)
Tao et al. [52]	Quantitative; Retrospective cohort	All patients presenting for care at a major STI clinic in Rhode Island between September 1, 2019—May 13, 2020 (n = 2347)	COVID-19	Service provision	• Compared to pre-COVID-19, there was a 55% (95% CI 45–63%; p < 0.001) reduction in the total number of STI clinic visits overall during COVID-19 lockdowns. More specifically, the number of screening visits were reduced by 60% (95% CI 46–71%; p < 0.001), provider visits by 50% (95% CI 35–62%; p < 0.001) and treatment visits by 62% (95% CI 40–75%; p < 0.001, when compared with the pre-COVID-19 • After lockdowns eased, there was an 84% (95% CI 68–88%, p < 0.001) reduction in total clinic visits, 100% reduction in screening visits, 68% (95% CI 56–77%, p < 0.001) reduction in provider visits, and 77% (95% CI 61–86%, p < 0.001) reduction in treatment visits compared to pre-COVID-19 phase
Tschann et al. [33]	Quantitative; Longitudinal	Health facilities that provide medication abortion across the US between April -October 2020 (n = 74)	COVID-19	Abortion	• In February 2020, 71% required 2 or more patient visits for a medication abortion. By April 2020, 35% reported reducing the total number of in-person visits associated with a medication abortion, and as of October 2020, 37 sites indicated newly adopting a practice of offering medication abortion follow-up with no in-person visits
White et al. [36]	Quantitative; Retrospective cohort	Abortion clinics in Texas (n = 18)	COVID-19	Abortion	• The number of abortions decreased by 38.0% (95% CI − 40.8% to − 35.1%) in April 2020, compared to April 2019 • The number of medication abortions increased accounting for 39% of all abortions in April 2019 to 80% in April 2020 • Texas residents receiving care at out-of-state facilities increased from 157 in February 2020 to 947 in April 2020 • After the COVID-19 related executive order was lifted in May 2020, the number of procedural abortions increased by 82.6% (95% CI 46.7%-127.4%)
Yuksel et al. [44]	Quantitative; Cross-sectional	Married patients who were older than 18 years and not menopausal (n = 58) in Turkey	COVID-19	Sexual behavior	• Sexual desire and frequency of intercourse significantly increased during the COVID‐19 pandemic, whereas quality of sexual life significantly decreased • Compared with 6–12 months, the pandemic is associated with increased sexual intercourse (2.4% vs 1.9%), decreased desire for pregnancy (32.7% vs 5.1%), decreased female contraceptive use (24% vs 10%), increased menstrual disorders (27.6% vs 12.1%) and lower FSFI (sexual function) scores (20.5 vs 17.6)

*COVID-19* coronavirus disease 2019, *STI* sexually transmitted infection; *FSFI* Female Sexual Functioning Index; *SARC* short-acting reversable contraceptive; *LARC* long-acting reversible contraceptive

#### Abortion

The majority of abortion-related studies report results exclusively from the US (n = 6, 75%). Across the US, the overall number of abortions decreased, however, demand for self-managed medication abortions increased during pandemic-related lockdowns and in the period immediately following lockdown. This was especially pronounced in states with greater stay-at-home orders, or in states with more restrictive abortion policies [32]. The need for in-person visits for medication abortion decreased from two visits among most providers (71%) to no in-person visits among 50% of abortion providers surveyed across the US [33]. Telemedicine, with in-person medication pick up or mail order was found to be acceptable during the pandemic in Hawaii, and was found to have high rates of success, follow-up retention and very little complications [34]. In a separate study of 103 abortion clinics across the US, several providers reported having to postpone, cancel or temporarily close their clinics due to staff being sick with probable COVID-19, COVID-19 related travel restrictions, or caregiving responsibilities, especially in the Southern states [35]. In Texas, an executive order postponing all unnecessary medical procedures (including abortion) prohibited most abortion procedures. Consequently, the total number of abortions fell by 38%, the number of out of state abortions increased by over 500%, and medication abortions increased by 41% [36]. Similarly, the number of medication abortions peaked at the height of COVID-19 in other states with restrictive abortion policies (Ohio, Kentucky & West Virginia) [37].

Similarly, the number of surgical and medication abortions decreased globally, due to fear of COVID-19, lack of transportation and access to pharmacies. Moreover, countries with restrictive abortion policies reported fewer women accessing abortion services, and fewer policy changes deeming SRH as essential to increase access to abortion or contraceptives during the pandemic [38]. A study in Nepal reported decreased demand for abortions during COVID-19 lockdown, which later increased once lockdowns were eased. Women who did receive abortions came in at a later gestational period and reported living closer to a health facility [39].

#### Contraceptive access & utilization

All studies examining the impact of the COVID-19 pandemic and associated lockdowns on contraceptive access and utilization reported substantial decreases. Nearly all SRH-related clinicians, researchers and practitioners surveyed from 29 different countries reported that access to contraceptives and other SRH-related services decreased, primarily due to the prioritization of the pandemic response over SRH. A few respondents from high-income countries reported that the pandemic provided an opportunity to expand access to medication abortion, through telehealth services [38]. In the US, most family planning providers (91%) reported providing telemedicine services for contraceptive counseling and prescriptions throughout the pandemic, with over half (53%) making referrals to a minority of patients for in-person services for LARC insertion/removal, Depo-Provera injections or other contraceptive-related issues [40]. An ecological study using insurance data from a national database in France found that prescriptions of contraceptives and of ovulation indicators initially increased by 47% and 16% in the first 2-weeks of lockdown, but then substantially decreased. The decrease was sustained in the 4-weeks post-lockdown [41]. Pandemic related lockdowns contributed to a 20% decrease in contraceptive uptake in rural Mozambique. Once the lockdown was eased, however, contraceptive referrals by community health workers increased by 18%. Moreover, uptake increased by 47% among women who were not currently using contraceptives, and by 80% by women who did not have phone access, and were likely of lower socioeconomic status [42].

In Northern Italy, where the majority of respondents reported using short-acting reversible contraceptives (SARC), half of all women who were not married/co-habiting discontinued their SARC during COVID-19 [43]. Of these, one-third reported an unintended pregnancy and sought an abortion. In Turkey, respondents reported a 14% decrease in contraceptive use, despite decreased desire for pregnancy, and increased sexual intercourse and menstrual disorders during COVID-19 [44]. In China, 9% of young women reported experiencing a shortage in contraceptives [45]. Similarly, few women (9%) reported difficulties accessing contraceptives in Australia, however, nearly a quarter (22%) reported unmet SRH-related needs, which include needing to access general practice, SRH specialist providers, pharmacies, hospitals, or counseling services [46]. In Nepal, 48% of women seeking safe abortion services reported an increased need for contraception, with 23% not using contraceptives due to inaccessibility because of lockdowns [39]. The type of contraceptive was not noted in these studies, however.

Only one study reported racial/ethnic disparities in contraceptive access. In the US, Black/African American, Latinx and Multi-racial respondents reported experiencing greater housing, transportation and food insecurity, when compared to White respondents. Poverty related factors of housing, transportation and food insecurity were found to be associated with an 86% greater difficulty in accessing contraceptives [47].

#### Menstruation

Only one study explicitly examined menstrual cycle changes [48], with almost half of all respondents reporting missed periods, with decreases and higher variability in cycle length. Yuksel et al. similarly report a 16% increase in menstrual disorders among survey respondents in Turkey [44]. The reason for menstrual cycle changes was not reported in either study, and it is not clear whether these changes were due to pandemic related lockdowns or COVID-19 infection.

#### Service provision

All studies noted that COVID-19 control measures resulted in decreased service provision and/or utilization. A study of providers from sexual health clinics across the US indicated that abortion services, HIV and STI testing decreased by 76%, 75% and 82%, respectively [49]. Consequently, telehealth services expanded, and provided greater access to services such as STI treatment based on symptomology and self-managed abortion. Using difference-in-difference analyses, Aiken et al. [32] found that requests for self-managed abortions increased during COVID-19, especially in states with greater stay-at-home behaviors, restrictions on in-clinic abortions, and/or those with especially high rates of COVID-19 incidence. Dell’Utri et al. compared obstetric and gynecological (OB/GYN) emergency service admissions during the COVID-19 pandemic, to the same period the year prior and found that overall admissions decreased by over 35% [50]. This translated to reduced admissions for complications related to pregnancy and gynecology. Similarly, Rimmer et al. (2020) reported changes to OB/GYN service provision in response to the COVID-19 pandemic in the UK [51]. These changes included reduced in-person antenatal care, elective procedures (such as fertility treatments or urogynecology), and inpatient stays. Patient-level outcomes were not reported, however. In China, women reported difficulties in accessing antenatal and/or maternal care; and obtaining appointments or medications for abortion services and STI testing [45]. Compared to pre-COVID-19 related lockdowns, the total number of clinic visits for STI screening, provider appointments and treatment decreased by 55%, and 84%, respectively at a STI clinic in Rhode Island during and after COVID-19 lockdown [52]. It is unclear how much of this decrease is attributed to pandemic-related lockdowns, fear of exposure, or decreased incidence of STIs due to decreased sexual activity. Disparities in service provision and/or utilization are unknown, however, as results are not described by sociodemographic status. Moreover, little is known about the impact of COVID-19 on fertility treatments and gynecological cancer screenings and treatment. Only one study, from Australia, reported that several women trying to conceive had actively stopped trying or were unable to continue because their in-vitro fertilization appointments had been cancelled [46].

#### Sexual behavior

Several studies examined changes in sexual behaviors and functioning during the COVID-19 pandemic. Married women in Turkey reported increased sexual desire and frequency of intercourse, but lower sexual functioning and quality of sexual life based on the Female Sexual Function Index (FSFI) [44]. Fuchs et al. [53] also used the FSFI to examine sexual functioning among women of reproductive age in Poland and found that overall sexual functioning and each FSFI domain (desire, arousal, lubrication, orgasm, satisfaction, and pain) decreased significantly, and sexual dysfunction doubled. In this study, women of lower socioeconomic status experienced lowest sexual functioning. Moreover, frequency of sexual activity declined due to isolation, conflict with partners and mental health. Decreases in sexual activity also included decreases in risky sexual behaviors in China [45]. Partner conflict was explicitly examined in the US, where one-third (34%) of all participants reported some degree of COVID-19 related conflict. An inverse dose–response relationship was observed between relationship conflict, sexual activity and other intimate behaviors [54].

#### Pregnancy intentions

In examining pregnancy intentions, a study in Turkey reported a 28% decline in pregnancy desire among women of reproductive age as result of the COVID-19 pandemic [44]. In Italy, 18% of respondents intended on getting pregnant before the pandemic, however, over one-third abandoned their intention due to future economic difficulties and further straining the healthcare system. In contrast, some respondents who did not intend to conceive reported doing so because of a need for positivity [55]. In Australia, most women surveyed indicated that they were trying to avoid pregnancy, and that the pandemic had not changed their pregnancy intentions. In the US, survey respondents indicated that the pandemic exacerbated housing and food insecurity among racial/ethnic minorities, which was associated with a decreased desire for pregnancy by over twofold [47].

#### Risk of bias

Overall studies were of low quality with scores ranging from 13 to 37 (40–88%) and averaging 23 (56%) points across all 24 eligible studies (Table 3). Of the 16 QATSDD items, the highest scoring items were a specific statement of aims/objectives (item 2); a clear description of the research setting (item 3), and fit between research question and analysis method (item 12). On average, lowest scoring items included the use of an explicit theoretical framework (item 1), statistical assessment of reliability and validity of measurement tool(s) (item 9), and evidence of user involvement in design (e.g., pilot study, informed by persons with lived experience, etc.; item 15). Although theoretical frameworks were not explicitly included, most authors stated why their research question was important within their given context. Items that did not score well may have been due to study design (e.g., observational vs randomized control trials). Similarly, statistical assessment of reliability and validity of measurement tool(s) may not have been considered due to lack of time for test re-test sampling, or the lack of validated tools measuring SRH-related outcomes. Finally, user involvement may not have been feasible or ethical during an epidemic.

**Table 3:** Quality assessment of eligible studies

Author	Item 1	Item 2	Item 3	Item 4	Item 5	Item 6	Item 7	Item 8	Item 9	Item 10	Item 11	Item 12	Item 13	Item 14	Item 15	Item 16	Total score	%	Rating
Aiken et al. [32]	0	3	3	0	3	3	2	2	1	3	N/A	3	3	N/A	0	1	27	64	High
Aryal et al. [39]	0	2	3	0	1	3	3	1	0	2	N/A	2	2	N/A	0	2	21	50	Low
Coombe et al. [46]	0	3	2	0	1	1	1	1	0	1	N/A	2	2	N/A	0	3	17	40	Low
Endler et al. [38]	0	2	1	0	1	1	1	1	0	2	N/A	1	1	N/A	1	1	13	31	Low
Fuchs et al. [53]	1	2	2	0	3	2	3	1	0	1	N/A	2	1	N/A	1	2	21	50	Low
Leight et al. [42]	0	3	3	1	3	3	3	1	0	3	N/A	3	3	N/A	0	3	29	69	High
Li et al. [45]	1	3	1	0	3	1	3	1	0	1	N/A	2	2	N/A	0	3	21	50	Low
Luetke et al. [54]	1	3	3	0	3	3	3	3	0	3	N/A	3	3	N/A	0	3	31	74	High
Nagendra et al. [49]	0	3	3	0	2	3	2	3	0	3	N/A	2	1	N/A	1	1	24	57	Low
Phelan et al. [48]	0	2	1	0	1	2	1	1	0	1	N/A	2	2	N/A	0	3	16	38	Low
Rimmer et al. [51]	0	3	3	0	1	3	3	3	0	3	N/A	3	2	N/A	2	3	29	69	High
Roberts et al. [35]	0	3	3	0	2	2	2	2	0	2	N/A	3	2	N/A	0	3	24	57	Low
Roland et al. [41]	0	3	3	3	3	3	3	1	0	3	N/A	3	3	N/A	0	0	28	67	High
Stifani et al. [40]	0	3	2	0	1	2	2	2	0	1	N/A	2	2	N/A	0	3	20	48	Low
Tao et al. [52]	0	3	3	0	3	2	3	1	2	3	N/A	2	2	N/A	0	3	27	64	High
Tschann et al. [33]	0	3	1	0	1	2	2	1	0	2	N/A	2	2	N/A	0	2	18	43	Low
White et al. [36]	1	3	2	2	3	2	3	1	2	3	N/A	3	3	N/A	0	2	30	71	High
Yuksel et al. [44]	1	3	3	3	3	3	3	3	3	3	N/A	3	3	N/A	0	3	37	88	High
Aiken et al. [32]	0	3	3	0	3	3	2	2	1	3	N/A	3	3	N/A	0	1	27	64	High
Aryal et al. [39]	0	2	3	0	1	3	3	1	0	2	N/A	2	2	N/A	0	2	21	50	Low
Caruso et al. [43]	1	3	3	0	3	1	1	3	0	2	N/A	1	0	N/A	0	1	19	45	Low
Coombe et al. [46]	0	3	2	0	1	1	1	1	0	1	N/A	2	2	N/A	0	3	17	40	Low
Dell'Utri et al. [50]	1	2	3	0	3	3	3	3	0	3	N/A	2	1	N/A	1	1	26	62	High
Endler et al. [38]	0	2	1	0	1	1	1	1	0	2	N/A	1	1	N/A	1	1	13	31	Low

Quality Assessment Tool for Studies with Diverse Designs [29]

Item 1: Explicit theoretical framework

Item 2: Statement of aims/objectives in main report

Item 3: Clear description of research setting

Item 4: Evidence of sample size considered in terms of analysis

Item 5: Representative sample of target group of a reasonable size

Item 6: Description of procedure for data collection

Item 7: Rationale for choice of data collection tool(s)

Item 8: Detailed recruitment data

Item 9: Statistical assessment of reliability and validity of measurement tool(s) (Quantitative studies only)*

Item 10: Fit between research question and method of data collection (Quantitative studies only)*

Item 11: Fit between research question and format and content of data collection tool e.g., interview schedule (Qualitative studies only)*

Item 12: Fit between research question and method of analysis

Item 13: Good justification for analytic method selected

Item 14: Assessment of reliability of analytic process (Qualitative studies only)*

Item 15: Evidence of user involvement in design

Item 16: Strengths and limitations critically discussed

Scores: 0 = not at all; 1 = very slightly; 2 = moderately; 3 = complete

Total scores > 60% = High quality; scores ≤ 60% = Low quality [31]

### Discussion

Beyond COVID-19 morbidity and mortality, all women and girls, including underserved populations, racial/ethnic or sexual minorities, immigrants and those with intersectional identities, will experience immediate and long-term consequences to their sexual and reproductive health [2, 12, 13]. Results from this review suggest that the indirect impact of the COVID-19 pandemic on SRH include significant reductions in access to abortion, contraceptives, and OB/GYN service provision. All studies reported in this review indicate that the total number of abortions decreased during the pandemic, but it is not clear whether this is due to decreased access because of pandemic-related lockdowns and SRH not being deemed an essential service, or due to decreases in sexual activity and changes in pregnancy intentions, as reported by some studies. Among women receiving abortions, the number of medication abortions increased, whereas surgical abortions decreased. Studies reported innovations such as telemedicine with or without in-person follow up visits for medication abortions, which were deemed safe, accessible and without complications [56]. The studies include in this review did not examine abortion access by sociodemographic characteristic or socioeconomic status, and disparities or inequities are unknown. The lack of unified abortion or epidemic control policies in response to COVID-19, however, likely widened existing health inequities [57].

Based on this review, COVID-19 pandemic related disruptions to family planning services were reported to decrease access to contraceptives, prescriptions, and/or uptake globally. This may be an unintended consequence of prioritizing COVID-19 response over SRH needs, and it is unclear how changes in sexual behavior and pregnancy intentions impact contraception uptake. Most studies included in this review did not report contraception method. The limited number of studies that did report contraception method suggest that service disruptions disproportionately impact women who rely on SARCs, as LARCS have been proven to be effective past their intended duration [58]. Of concern is that the reduction in contraceptive use was sustained, even once lockdowns were eased in some places [41]. On the other hand, some places showed a promising rebound, especially among women who were not using contraceptives previously and women of lower socio-economic status [42]. Only one study examined how poverty related factors are negatively associated with contraceptive access in the US [47].

Our search returned no studies on the impact of other respiratory epidemics on women and girls SRH outcomes (not including pregnancy and birth-related outcomes, gender-based violence, and maternal and child health). This may be because the prioritization of epidemic response has overshadowed SRH, the historic lack of investment in SRH, or the dismissal of SRH as rooted in structural gender inequities. However, given the rise in emerging infectious diseases [59] and increasing calls for attention to SRH during pandemics/epidemics [2, 12, 13], this finding emphasizes the need to examine the full range of SRH outcomes, that is inclusive of HIV/STIs; comprehensive sexuality education; safe abortion; prevention, detection, and counselling for gender-based violence; prevention, screening and treatment of infertility and gynecological cancers; and counseling and care for sexual health and well-being [60]. High quality evidence of the indirect, downstream consequences of epidemics is needed to inform future policy and planning, ensure SRH equity, and generate equitable access to the full range of SRH services. Moreover, few studies included in this review examined the indirect impact of COVID-19 and the pandemic response on SRH outcomes among underserved populations, racial/ethnic or sexual minorities, immigrants, or those with intersectional identities. The pandemic has been found to exacerbate poverty, disproportionately impact people of lower socioeconomic status, and aggravate existing health issues, including those related to SRH [61]. Interventions are critically needed to sustain adequate access to abortion, family planning, STI/HIV testing and treatment, ensure continuity of fertility treatments, gynecological cancer screenings and treatment, and other SRH service provision, especially among women of lower socioeconomic status, to reduce the number of unintended pregnancies, unsafe abortions, STI/HIV transmission, and halt the decades of progress made on health and development [62].

SRH is a human right that is vital for sustainable development, and should be among the least restrictive solutions in the context of epidemic control [2, 13, 63]. Historically, restrictive SRH policies have perpetuated inequities among Black, Latinx, and immigrant women, and are expected to widen as a result of COVID-19 and related policies for epidemic control. Few studies of respiratory epidemics and SRH explicitly examined outcomes among women with diverse lived experiences, despite the accumulating evidence that indicate that COVID-19 disproportionately impacts racial/ethnic minorities, immigrants, and people with lower socio-economic status [12, 64]. This review highlights a gap in research of SRH service provision, access and utilization among marginalized groups of women and girls and those with intersectional identities, including adolescent and young girls, those with disabilities, sexual or ethnic/racial minorities, refugees and immigrants, many of whom experience difficulties in accessing SRH services notwithstanding an epidemic [10, 11, 13]. Several studies noted the expansion of telehealth services, offers an opportunity to reach more women and girls, including those traditionally underserved. Yet, little research on how telehealth has improved or constrained SRH access for underserved populations has been published. This review highlights the need to understand the indirect impact of COVID-19 and its control measures on the wider range of SRH outcomes and populations of women and girls in the long-term.

This study is not without limitations. Given the rapid timeline of this review and the constantly evolving research on COVID-19, we included readily available studies on COVID-19 impacts on SRH at the time of review, but new findings emerge on a weekly basis. Moreover, there were no restrictions on location, and generalizability of results may be inadequate due to variations in epidemic control policies. Non-respiratory epidemics (e.g., Ebola, HIV, Zika, etc.) have also impacted SRH outcomes; however, these were not included as modes of transmission and infection control measures varied too greatly. Although we apply a reproductive justice lens, we excluded maternal and child health outcomes beyond pregnancy, childbirth and violence. While these are an important aspect of reproductive justice, this literature seemed to be its own body of work and dedicated systematic reviews have been published elsewhere [17–28]. Finally, the majority of studies included in this review were of low quality; possibly because the majority of studies reported in this review were observational, and more rigorous research on the impact of pandemics on SRH is needed. Randomized-control trials are the gold-standard for high-quality studies, however, they are not always feasible, practical or ethical within the context of an infectious disease epidemic, and studies without a comparison group should be interpreted with caution. Conversely, quasi-experimental designs are useful in determining causal relationships when randomized control trials cannot be used for practical or ethical reasons [65]. The COVID-19 pandemic provides an opportunity to use quasi-experimental designs to better understand the indirect impact of COVID-19 and the pandemic response on SRH outcomes among marginalized women and girls. Future research using quasi-experimental designs are needed to provide robust evidence of the impact of interventions and/or policy changes (e.g., telemedicine with in-person versus mail order medication abortion, executive orders that did not deem SRH as an essential service, etc.) on SRH-related outcomes.

By being aware of the impacts of COVID-19 on SRH, policy makers can be better prepared to enact guidelines and policies that promote reproductive justice and access to equitable, timely SRH, despite lockdowns. Given the service disruptions evident in this review, providers should prioritize education and provision of various contraceptive methods, and when appropriate, should counsel and allow patients to consider switching methods. Patient education on the range of contraceptive methods, protocols for switching methods, at-home use of contraceptive methods (including injectables [66]) and self-managed abortion can be completed via telehealth, which may provide an opportunity to reach more women and girls. Although not included in this review, COVID-19 has resulted in notable increases in gender-based violence and reproductive coercion [28, 67], making access to contraceptives and abortion services vital for ensuring access to care and reproductive justice.

Changes to service provision, in response to COVID-19, must consider historical inequities in access to SRH services. Clear and consistent guidelines for changes to service provision that ensure access to quality SRH services are needed. Moreover, increased efforts should be made to collect sociodemographic information to better understand the indirect and downstream impact of the COVID-19 pandemic on SRH for diverse groups. Finally, while the expansion of telehealth services provides an opportunity to reach underserved populations, privacy concerns, disparities in access to technology, and longstanding impacts of racism on care uptake must also be considered.

## Conclusion

As COVID-19 presents new challenges to accessing essential SRH services, the application of a reproductive justice lens is crucial to ensure SRH inequities do not continue to widen. Evidence suggests that COVID-19, and its control measures disproportionately impact women’s SRH outcomes. Results indicate that OB/GYN and SRH service provision, pregnancy intentions and sexual behavior, access to family planning, contraceptives and abortion markedly decreased, as an indirect consequence of the COVID-19 pandemic response. Accumulating evidence indicates that COVID-19 disproportionately impacts marginalized and underserved populations directly, yet these are the groups least represented in the research. More research dedicated to the diverse lived experiences of women and higher quality evidence is needed to prevent and mitigate the indirect impact of COVID-19 and lockdown measures on long-term SRH outcomes.

## Data Availability

Not applicable.
